# Effect of Mo on Microstructures and Wear Properties of In Situ Synthesized Ti(C,N)/Ni-Based Composite Coatings by Laser Cladding

**DOI:** 10.3390/ma10091047

**Published:** 2017-09-06

**Authors:** Fan Wu, Tao Chen, Haojun Wang, Defu Liu

**Affiliations:** 1 College of Mechanical and Electrical Engineering, Central South University, Changsha 410083, China; wufan1993@csu.edu.cn (F.W.); chent0731@csu.edu.cn (T.C.); wanghaojun@csu.edu.cn (H.W.); 2 State Key Laboratory of High Performance Complex Manufacturing, Changsha 410083, China

**Keywords:** laser cladding, in situ synthesis, composite coating, titanium alloy, wear resistance

## Abstract

Using Ni60 alloy, C, TiN and Mo mixed powders as the precursor materials, in situ synthesized Ti(C,N) particles reinforcing Ni-based composite coatings are produced on Ti6Al4V alloys by laser cladding. Phase constituents, microstructures and wear properties of the composite coatings with 0 wt % Mo, 4 wt % Mo and 8 wt % Mo additions are studied comparatively. Results indicate that Ti(C,N) is formed by the in situ metallurgical reaction, the (Ti,Mo)(C,N) rim phase surrounding the Ti(C,N) ceramic particle is synthesized with the addition of Mo, and the increase of Mo content is beneficial to improve the wear properties of the cladding coatings. Because of the effect of Mo, the grains are remarkably refined and a unique core-rim structure that is uniformly dispersed in the matrix appears; meanwhile, the composite coatings with Mo addition exhibit high hardness and excellent wear resistance due to the comprehensive action of dispersion strengthening, fine grain strengthening and solid solution strengthening.

## 1. Introduction

Titanium alloys are widely applied in industrial areas, such as the aerospace and automotive industries, because of the high specific strength and exceptional corrosion-resistant properties. Nevertheless, the undesirable characteristics of low hardness and poor tribological properties seriously prevent titanium alloys from being used in friction scenarios [1,2,3]. As is well known, the fabrication of a wear-resistant coating on titanium alloy is one of the best methods to improve the wear-resistance [4]. As an advanced surface modification technology, laser cladding is widely employed to prepare various functional coatings on metals and their alloys [5,6,7,8]. It has also been proven that metal matrix composite (MMC) coatings fabricated by laser cladding can effectively enhance the tribological properties. Li et al. [9] created laser-cladded TiC/Ti_3_Al–TiAl composite coatings on Ti6Al4V alloys, and the wear properties of the coatings were approximately increased by twice in comparison with the substrate. Weng et al. [10] obtained in situ formed Ti_5_Si_3_–TiC/Co-based composite coatings using SiC + Co42 alloy powders by the laser cladding process, and the anti-wear properties of the coatings were 18.4~57.4 times higher than that of the titanium alloys. In addition, TiB–TiC/Ti_2_Ni-α(Ti) [11] and WC/Ni [12] coatings with better wear-resistance than the titanium alloys were also investigated.

In order to fabricate high wear-resistant coatings, suitable coating materials should be chosen. Self-fluxing alloy (Fe-based, Ni-based, Co-based alloys, etc.) powders have been used as laser cladding materials in virtue of the characteristics of low melting point and good wettability [13]. Moreover, previous investigations indicated that the composite materials composed of self-fluxing alloy powders and hard ceramic particles can fabricate coatings with a better microstructure and better mechanical properties [14,15,16]. As the solid solution of TiC and TiN, Ti(C,N) ceramics exhibit superior properties of high hardness and superb wear resistance and are often regarded as ideal reinforced phases. Yang et al. [17] fabricated TiCN/Ti coatings by the laser cladding of TiCN + Ti alloy powders on titanium alloys, and the wear properties of coatings were 4.2 times higher than that of the substrate. According to the report of Li et al. [18], the laser-cladded TiC–TiN–TiCN/Al_3_Ti–Ti_3_Al intermetallic coatings showed a significant increase in wear-resistant properties (4.5~5 times) compared with the Ti6Al4V alloys. Furthermore, previous studies indicated that Mo or Mo_2_C played an important role in improving the wettability between TiC or Ti(C,N) and Ni matrix; meanwhile, cermets with Mo or Mo_2_C addition showed a finer microstructure and better mechanical properties [19,20,21,22]. However, investigations on the influence of Mo on the microstructures and wear properties of the laser-cladded Ti(C,N)/Ni coatings have been rarely involved so far.

In this paper, in situ synthesized Ti(C,N)/Ni composite coatings are fabricated on Ti6Al4V alloys using Ni60 + C + TiN mixed powders as the laser cladding materials, and the microstructures and wear-resistance of the coatings containing different Mo contents are investigated.

## 2. Materials and Methods

The substrates are the Ti6Al4V alloy plates, which are cut into specimens with dimensions of 30 m × 30 mm × 6 mm. The precursor powders are composed of Ni60 (particle size 40–100 µm, ≥99.5% purity), C (particle size 10–20 µm, ≥99.9% purity), TiN (particle size 1–10 µm, ≥99.9% purity) and Mo (particle size 1–10 µm, ≥99.9% purity). The chemical compositions (mass fraction, %) of the Ni60 alloy powders are listed in Table 1.

All specimens for laser cladding are polished by SiC grit paper (240#) and degreased with alcohol and acetone. The precursor powders are evenly mixed in a planet ball miller for 3 h, and preplaced on the surfaces of the specimens with approximately 0.6 mm in thickness. After dried in an oven at the temperature of 100 °C for 2 h, the preplaced coatings are melted by RFL-C500 fiber laser with average power of 500 W, and the laser cladding process is protected by argon gas with a flowing rate of 10 L/min. It is reported that excessive Mo content will increase the thickness of the rim phase and lead to coarser ceramic phase grains, which means that the mechanical properties of Ti(C,N)/Ni cermets will decrease [19,20]. Moreover, adding excessive high-melting-point Mo will significantly shorten the surviving life of the laser molten pool so that there is not enough time for the air to escape from the molten pool, which produces pores, cracks and other defects in the coating. Hence, the content of Mo in the preplaced powders should be controlled. The compositions of preplaced powders and the optimized laser cladding parameters are listed in Table 2. In this paper, the cladding coatings containing 0 wt %, 4 wt % and 8 wt % Mo are respectively referred to as 0Mo, 4Mo and 8Mo coating on the basis of the powder compositions.

The cross-sectional metallographic samples of the cladding coatings are polished, and then etched in a HNO_3_ + HF + H_2_O solution (with a volume rate of HNO_3_:HF:H_2_O = 2:3:5) for 1 min. The microstructure morphologies of the samples are observed by QUANTA FEG 250 scanning electron microscope (SEM, FEI, Hillsboro, OR, USA) with an energy-dispersive spectrometer (EDS, Oxford Instruments, Oxford, UK). The phase compositions of the coatings are investigated by D/500 X-ray diffractometer (XRD, Bruker, Berne, Switzerland). The micro-hardness distributions of the coatings are tested by HVS-1000Z micro-sclerometer (Vegour, Shanghai, China) with a 0.2 kg load and 15 s loading time. 

Before the dry friction tests at room temperature, the multi-track coatings with 40% overlap rate are abraded on SiC grit papers with grit mesh sizes from 240# to 800#. HT-1000 friction and wear tester is used to examine the wear properties of the coatings with a load of 20 N. The rotational speed, the friction diameter and the wear time are 400 rpm, 10 mm and 30 min, respectively. The grinding ball is made up of Si_3_N_4_ ceramic, and its diameter is 5 mm and hardness is 1700 HV. The wear rate is calculated with the following formula:

(1) $$W=\frac{V}{T}$$

where W is wear rate (mm^3^/min); *V* is total wear volume (mm^3^); *T* is total wear time (min).

## 3. Results and Discussion

### 3.1. Phase Constituent Analysis

**Figure 1:**
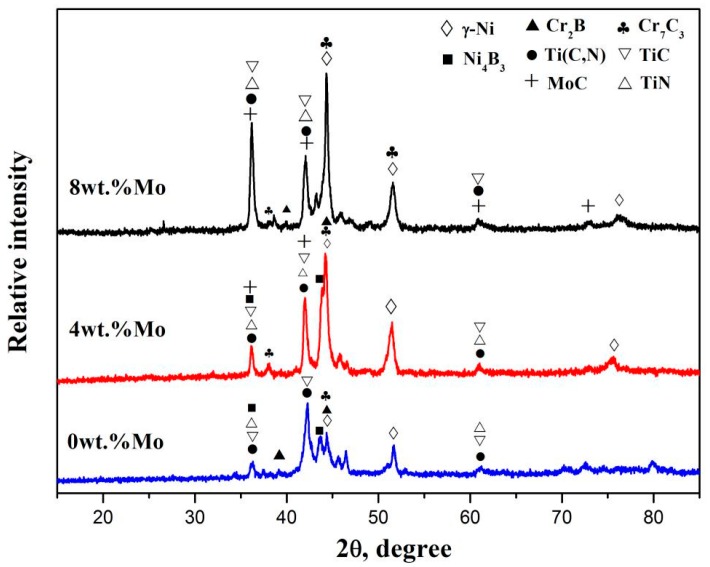
X-ray diffraction of coatings with different Mo contents.

The XRD results of the three different laser cladding coatings are presented in Figure 1. The 0Mo coating is mainly composed of γ-Ni solid solution, Ni_4_B_3_, Ti(C,N), TiC, TiN, Cr_2_B and Cr_7_C_3_. Under the irradiation of the laser beam, the preplaced materials and the surface layer of the substrate simultaneously melt. In this case, a large amount of Ti released from the Ti6Al4V alloy enters into the molten pool due to the dilution effect. Then complex chemical reactions occur in the molten pool, resulting in the formation of above phases, including TiC and Ti(C,N) [8,18]. During the laser cladding process, Ti in the molten pool reacts with C from preplaced powders, forming the TiC. Furthermore, in-situ synthesized TiC reacts with ex-situ TiN, and consequently Ti(C,N) is formed. The possible chemical reactions are described as follows:

Ti + C → TiC(2)

TiC + TiN → Ti(C,N)(3)

Besides this, Cr_2_B and Cr_7_C_3_ are also detected in the 0Mo coating owing to the reactions between Cr and B, C [13]. It has been noted that the 4Mo and 8Mo coatings contains not only all of the above phases, but also a new phase MoC, which is derived from the reaction between Mo and C. Meanwhile, the diffraction peak of MoC is higher in 8Mo coating than that of 4Mo coating, which is coincides with the increasing Mo content. 

### 3.2. Microstructure Analysis 

**Figure 2:**
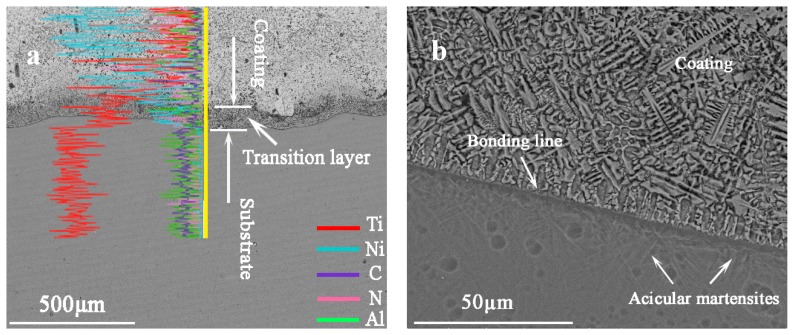
The cross-sectional morphology (**a**) and the energy dispersive spectrometer (EDS) composition changes of the 0Mo coating across interface (**b**).

The cross-section morphology and EDS composition changes of the 0Mo coating across the interface are presented in Figure 2, which is typical for all the cladding coatings. It can be seen from this figure that the coating is free of pores and cracks and is metallurgically bonded with the Ti6Al4V alloy. According to the line analysis result, partial Ti elements diffuse from the substrate into the coating, and a transition layer between the coating and the substrate is formed (Figure 2a). In addition, crystals in planar and cellular morphologies appear near the bonding line because of the ultra-high thermal gradient, as shown in Figure 2b. Due to the rapid melting and rapid solidification caused by laser cladding, acicular martensites (a′-Ti) [13] emerge in the substrate near the bonding line, which is regarded as heat-affected zone.

**Figure 3:**
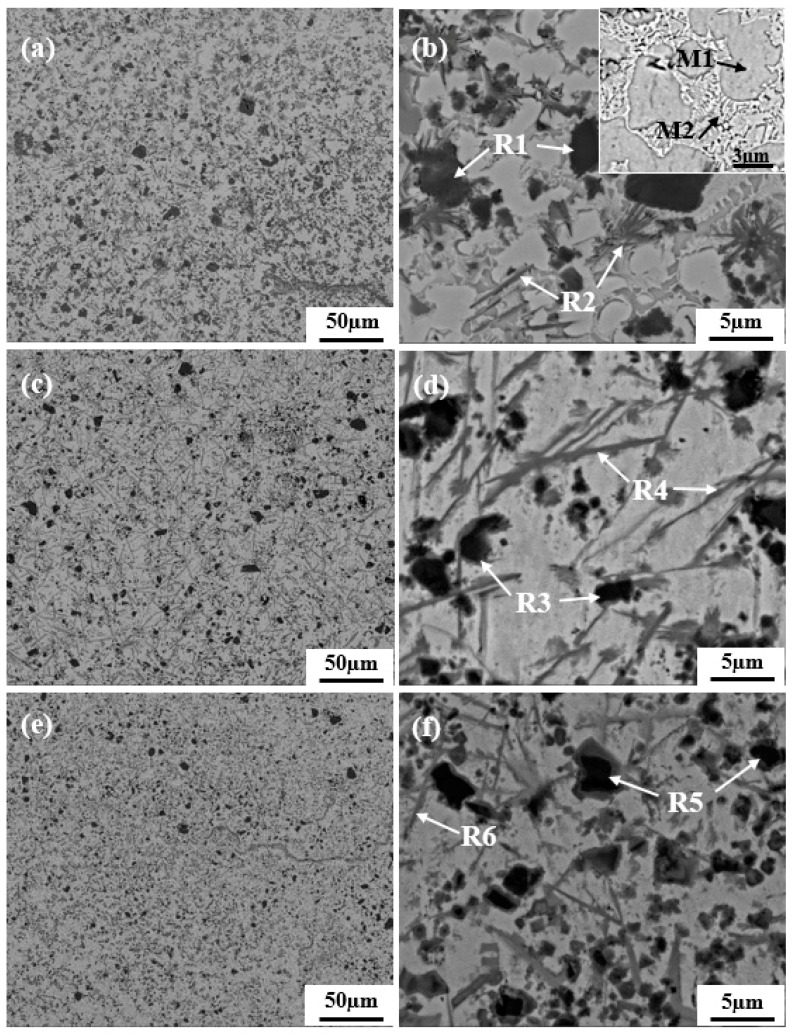
Microstructure morphologies of the middle section typically for: (**a**,**b**) 0Mo coating; (**c**,**d**) 4Mo coating; (**e**,**f**) 8Mo coating.

The microstructure morphologies of the middle sections in the coatings are illustrated in Figure 3. It can be seen from Figure 3a,b that numerous dark blocky phases (R1) and deep grey whisker phases (R2) are uniformly distributed in the light grey matrix that mainly consists of cellular grains (M1) and lamellar eutectics (M2) between the cellular grains. The EDS analysis results of the phases are shown in Table 3. From Table 3, it can be determined that cellular grains (M1) mainly consist of γ-Ni solid solution which contains Fe, Cr and C. The lamellar eutectics (M2) include not only Ni, Cr and C but also a small amount of B, and it can be identified as γ-Ni + Ni_4_B_3_ eutectics. The blocky phases (R1) mainly contain Ti, C and N, and can be identified as Ti(C,N) by referring to the XRD analysis. The whisker phases (R2) mainly consist of Cr, C and B, indicating the existence of Cr_7_C_3_ and Cr_2_B. The SEM images of the 4Mo and 8Mo coatings presented in Figure 3c,e reveal that the grain becomes finer and more homogeneous with the addition of Mo. The grain refinement is highly related to the dissolution–precipitation process. On the one hand, Mo can decrease the solubility of TiN or Ti(C,N) in the Ni matrix, and more small TiN particles are remained [23,24]. On the basis of the heterogeneous nucleation theory, the grain crystals tend to attach themselves on the already existed phases in order to reduce the energy for their growth [13]. In this case, the undissolved TiN will act as the nucleation site for the grain growth, so the grain is refined due to the increased number of crystal nuclei. On the other hand, Mo can improve the wettability between ceramic phases and matrix, and reduce the contact among ceramic phases, thus the aggregation and growth of ceramic phases are effectively restrained [21,22]. The enlarged images (Figure 3d,f) show that the microstructure of the 4Mo and 8Mo coatings mainly consists of dark blocky phases (R3 and R5) and deep grey whisker phases (R4 and R6), which is similar to that of the 0Mo coating. According to the EDS results and the XRD analysis, the blocky phases (R3 and R5) and the whisker phases (R4 and R6) are identified as Ti(C,N) and Cr_2_B/Cr_7_C_3_ respectively. In addition, an interesting phenomenon occurs when Mo is added into the cladding materials. It has been noted that the black Ti(C,N) is surrounded by a grey rim phase (as shown in Figure 3d,f), which is generally acknowledged as the core–rim structure. Simultaneously, with the increase of Mo content, the blocky Ti(C,N) particles are more regular in shape and the thickness of the rim phases increases.

**Figure 4:**
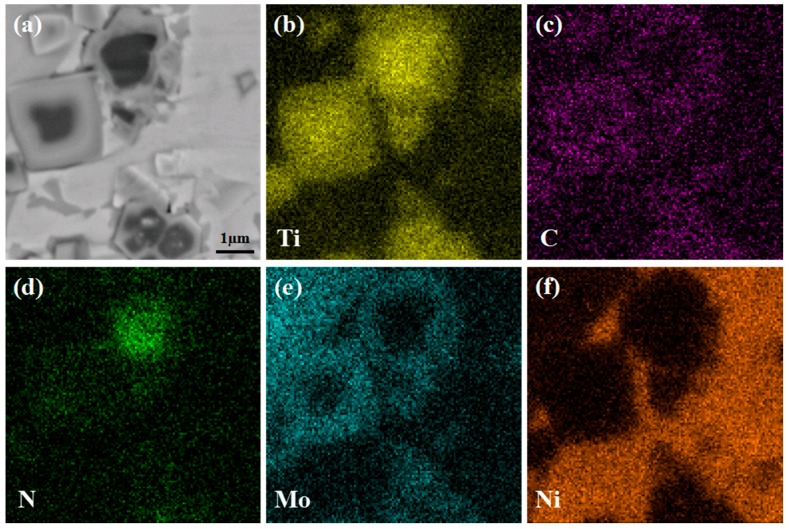
SEM micrograph (**a**) and corresponding elemental distributions (**b**–**f**) of core-rim structure in 8Mo coating.

The SEM micrograph at higher magnification and the corresponding elemental distribution results of the core–rim structure in the 8Mo coating are shown in Figure 4. It is indicated that the black core mainly consists of Ti and N, which is identified as the initial TiN. Moreover, Ti, C, N and Mo are detected synchronously in the grey rim phase, which is confirmed as (Ti,Mo)(C,N) [20,22]. In comparison, the Mo content in the grey rim phase is higher than that of the black core, so the rim phase presents a lighter color in SEM-BSE mode. Remarkably, the core-rim structure with a high Mo content in shell is of great significance to enhance the performance of Ti(C,N)/Ni composite coating. On one hand, the addition of Mo plays a role of solid solution strengthening in the matrix [21,22,23]; on the other hand, it can improve the wettability between Ti(C,N) ceramic and Ni matrix, forming finer microstructure [23,24]. 

**Figure 5:**
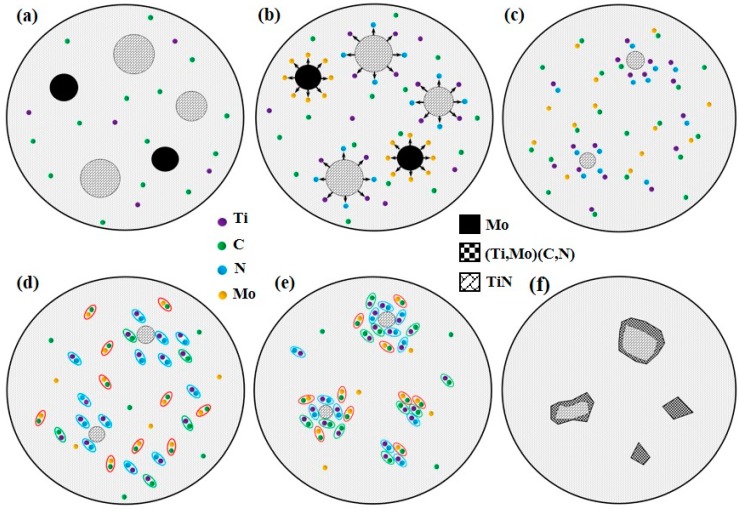
The diagram illustration for the formation mechanism of Ti(C,N) and the action mechanism of Mo: (**a**) the formation of initial molten pool; (**b**) dissolution of TiN and Mo; (**c**) partially melted large TiN particles; (**d**) the formation of TiN, TiC and MoC; (**e**) the formation and growth of Ti(C,N); (**f**) the effect of Mo on the Ti(C,N).

To explain the formation mechanism of the core–rim structure, a simplified diagram illustration is demonstrated in Figure 5. Under the irradiation of the laser beam, the preplaced powders absorb vast amounts of heat and Ni-based alloy powders with the lowest melting point (1027 °C) melt firstly, forming the initial molten pool (Figure 5a). With the temperature of the molten pool rising, TiN is decomposed into Ti and N atoms and Mo dissolves into the laser molten pool (Figure 5b). It is likely that small TiN particles completely melt, whereas some large TiN ceramics partly dissolve. Because of the stirring action of the convection current, the dissolved atoms are well-distributed in the laser molten pool (Figure 5c). Then, TiN is re-formed due to the chemical reaction between Ti and N atoms, and TiC and MoC are in-situ formed with the regeneration of TiN (Figure 5d). According to the crystal growth theory [13], the newly generated TiC and TiN tend to precipitate on the surface of the undissolved TiN to reduce the growth energy, and consequently form the Ti(C,N) solid solution. In addition, partial TiN and TiC probably nucleate independently and grow up due to the non-even constituent and heat distribution in the molten pool (Figure 5e). Ultimately, the in situ synthesized MoC precipitates onto the Ti(C,N) particles, which can significantly restrain the aggregation and growth of Ti(C,N). Moreover, investigations [20,25] showed the MoC and Ti(C,N) could diffuse into each other and form the (Ti,Mo)(C,N), which is the solid solution of MoC and Ti(C,N). For the large Ti(C,N) particles, the diffusion occurs only on the shells and the cores are still composed of TiN, forming the black core/grey rim structure, while the inter-diffusion is complete for the small Ti(C,N) particles and the whole Ti(C,N) grains are transformed into grey (Ti,Mo)(C,N) phases, which results in the disappearance of the rim phases (Figure 5f).

### 3.3. Micro-Hardness and Wear Resistance

**Figure 6:**
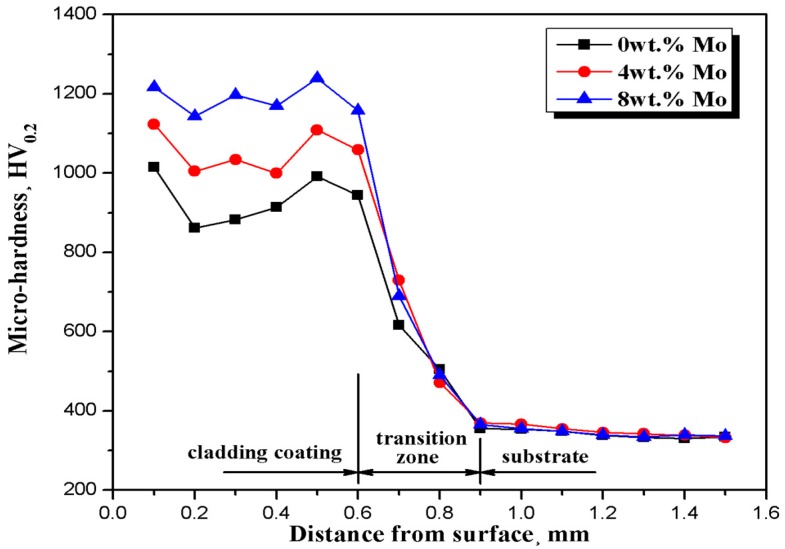
Micro-hardness profiles along the depth direction of the cladded coatings.

The micro-hardness profiles along the depth direction of the coatings are demonstrated in Figure 6. Although all the coatings have a similar micro-hardness distribution, the hardness of composite coatings presents a significant uptrend with the increasing Mo content. For 0Mo, 4Mo and 8Mo coating, the micro-hardness are respectively enhanced by 2.8, 3.1 and 3.5 times than that of the Ti6Al4V substrate (336.8 HV_0.2_). As mentioned above, the addition of Mo can not only refine the grain size, but also form new enhancing phases, such as MoC. Hence, due to the comprehensive action of fine-grain strengthening and dispersion strengthening, coatings with larger Mo proportion present higher hardness. Meanwhile, because Mo can suppress the aggregation of enhancing phases, the hardness distribution of coatings with a higher Mo content is more homogeneous. In this case, the 0Mo coating exhibits the sharpest fluctuation in hardness curve; whereas, the least variance is presented in the micro-hardness profile of 8Mo coating. 

**Figure 7:**
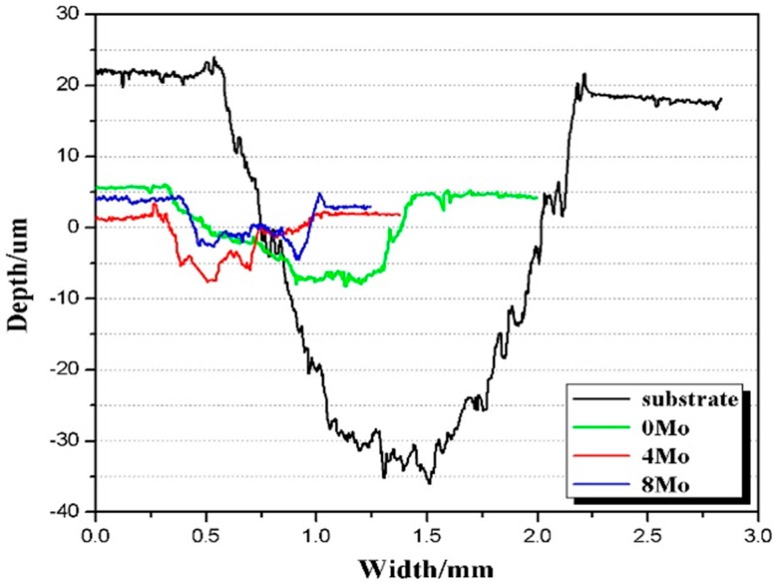
3D non-contact surface profiles across the wear scars of the substrate and the coatings.

**Figure 8:**
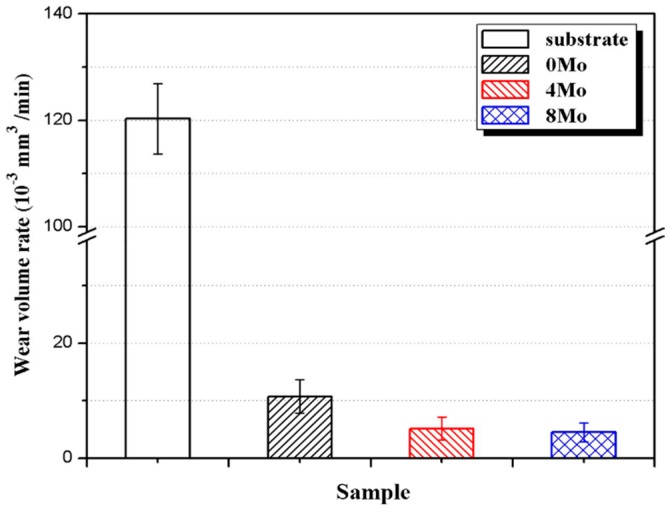
Wear volume rate of the substrate and the coatings.

The 3D non-contact surface profiles across the wear scars of the substrate and the coatings are shown in Figure 7. All cladding coatings present smaller wear scar width and depth than the Ti6Al4V alloy; and both the wear scar width and depth show downtrends with the increasing Mo content. The wear volume rates of the Ti6Al4V substrates and the coatings are presented in Figure 8, which indicates that the cladding coatings exhibit much better abrasion resistance. For the Ti6Al4V alloy, 0Mo, 4Mo and 8Mo coating, the wear volume losses are 120.28 × 10^−3^ mm^3^·min^−1^, 10.73 × 10^−3^ mm^3^·min^−1^, 5.14 × 10^−3^ mm^3^·min^−1^ and 4.50 × 10^−3^ mm^3^·min^−1^, respectively. In comparison with the substrate, the wear resistance of 0Mo, 4Mo and 8Mo coating is increased by 11.2, 23.4 and 26.7 times, respectively. This illustrates that coatings containing a higher Mo proportion present better wear resistance, which can be attributed to the effects of the higher micro-hardness. 

**Figure 9:**
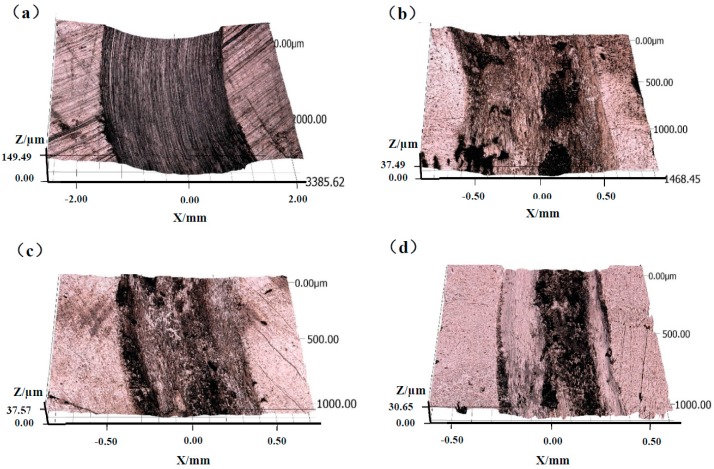
3D non-contact surface mapping of the wear scars of (**a**) substrate; (**b**) 0Mo coating; (**c**) 4Mo coating and (**d**) 8Mo coating.

**Figure 10:**
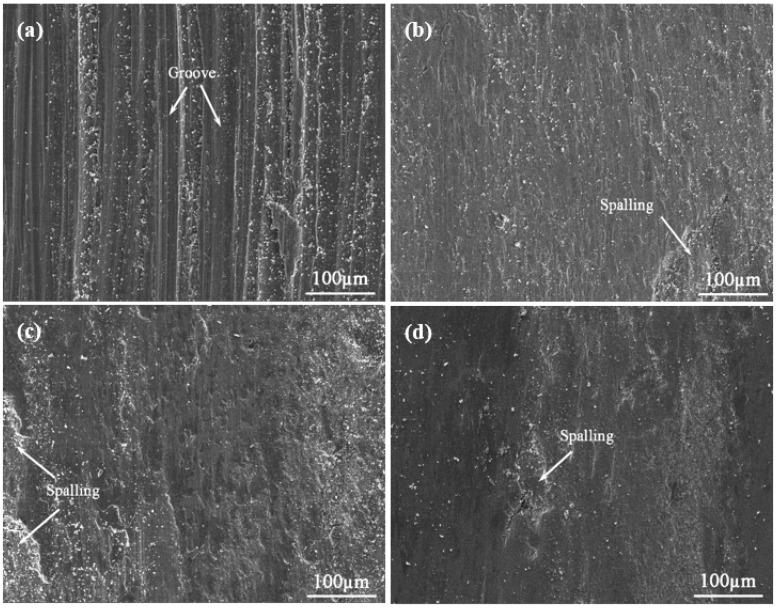
Wear morphologies of (**a**) substrate; (**b**) 0Mo coating; (**c**) 4Mo coating and (**d**) 8Mo coating.

The wear morphologies of the cladding coatings and the substrate present different characteristics, as shown in Figure 9 and Figure 10. It is observed from Figure 9a and Figure 10a that there are deep grooves and adhesive features on the wear surface of the Ti6Al4V alloy, which indicates that the substrate suffers from abrasive and adhesive wear. During the process of dry sliding friction, it is easy for the hard asperities on the grinding ball to penetrate into the surface of relative soft titanium substrate, forming micro-cutting and deep grooves on the abrasive surface. By contrast, no obvious characteristic of micro-cutting or ploughing grooves is detected in the worn surfaces of the coatings due to the higher hardness of the coatings, and the wear mechanism of the composite coatings is mainly abrasive wear. Furthermore, the wear surfaces of the coatings become smoother with the increase of Mo, which is in consistent with the hardness of the coatings. As mentioned above, higher Mo content in coatings can result in finer microstructure, and in the meanwhile, the toughness of the coating is improved. Hence, the most obvious spalling characteristic appears in the 0Mo coating due to the worst toughness (Figure 9b and Figure 10b). However, the best toughness contributes to the least obvious spalling characteristic of the 8Mo coating (Figure 9d and Figure 10d). 

## 4. Conclusions

(1) In this paper, using Ni60 alloy, C, TiN and Mo powders as the precursor materials, in situ synthesized Ti(C,N) reinforced Ni-based composite coatings are produced on Ti6Al4V alloys by laser cladding. The composite coating is mainly composed of γ-Ni, Ni_4_B_3_, Ti(C,N), TiC, TiN, Cr_2_B and Cr_7_C_3_ phases.
(2) The microstructures and wear resistance of the cladding coatings with different Mo contents are analyzed contrastively. Results indicate that, with the addition of Mo, the microstructure of the coatings becomes finer and more homogeneous; meanwhile, a unique core–rim structure appears in the coating.
(3) The effect of Mo on the mechanical properties of composite coating is obvious. The hardness and wear resistance increase with the increase of Mo content. Compared with the titanium alloy, the wear resistance of the 0Mo, 4Mo and 8Mo coating is improved by 11.2, 23.4 and 26.7 times, respectively.

## Figures and Tables

**Table 1:** The chemical composition (mass fraction, %) of the Ni60 self-fluxing alloy powder.

Cr	Fe	W	Si	B	C	Ni
15.5	15	3	4	3.5	0.65	Bal.

**Table 2:** The compositions of the preplaced powders and optimized laser cladding parameters.

Number	Powder Composition (wt %)	Laser Power (W)	Scanning Speed (mm/s)	Spot Diameter (mm)
Sample 1	88Ni60-2C-10TiN	500		
Sample 2	84Ni60-2C-10TiN-4Mo		2	3
Sample 3	80Ni60-2C-10TiN-8Mo			

**Table 3:** The results of the EDS analysis (atom fraction, %).

Phase	Ti	N	C	Fe	Cr	B	Ni	Mo
M1	3.02	-	12.24	10.90	8.13	-	65.71	-
M2	3.99	-	15.45	2.49	7.29	8.66	62.12	-
R1	33.00	34.65	27.35		1.26	-	3.74	-
R2	1.63	-	17.60		27.92	50.03	2.82	-
R3	43.55	39.62	16.83		-	-	-	-
R4	0.92	-	27.82		19.07	44.90	6.92	0.37
R5	33.43	55.48	10.44		-	-	-	0.65
R6	2.70	-	25.26		17.26	42.96	8.04	3.78
